# High Glucose Concentrations Affect Band 3 Protein in Human Erythrocytes

**DOI:** 10.3390/antiox9050365

**Published:** 2020-04-27

**Authors:** Rossana Morabito, Alessia Remigante, Sara Spinelli, Giulia Vitale, Vincenzo Trichilo, Saverio Loddo, Angela Marino

**Affiliations:** 1 Department of Chemical, Biological, Pharmaceutical and Environmental Sciences, University of Messina, Viale F. Stagno D’Alcontres 31-98166, 98122 Messina, Italy; rmorabito@unime.it (R.M.); aremigante@unime.it (A.R.); sara.spinelli1992@libero.it (S.S.); giulia.vitale19@gmail.com (G.V.); 2 Department of Clinical and Experimental Medicine, AOU Policlinico Universitario “G. Martino”, Via Consolare Valeria-98125, 98124 Messina, Italy; vtrichilo@unime.it (V.T.); sloddo@unime.it (S.L.)

**Keywords:** diabetes, glucose exposure, oxidative stress, Band 3 protein, erythrocytes, SO_4_^2−^

## Abstract

Hyperglycemia is considered a threat for cell homeostasis, as it is associated to oxidative stress (OS). As erythrocytes are continuously exposed to OS, this study was conceived to verify the impact of either diabetic conditions attested to by glycated hemoglobin (Hb) levels (>6.5% or higher) or treatment with high glucose (15–35 mM, for 24 h) on erythrocyte homeostasis. To this aim, anion exchange capability through the Band 3 protein (B3p) was monitored by the rate constant for SO_4_^2−^ uptake. Thiobarbituric acid reactive species (TBARS), membrane sulfhydryl groups mostly belonging to B3p, glutathione reduced (GSH) levels, and B3p expression levels were also evaluated. The rate constant for SO_4_^2−^ uptake (0.063 ± 0.001 min^−1^, 16 min in healthy volunteers) was accelerated in erythrocytes from diabetic volunteers (0.113 ± 0.001 min^−1^, 9 min) and after exposure to high glucose (0.129 ± 0.001in^−1^, 7 min), but only in diabetic volunteers was there an increase in TBARS levels and oxidation of membrane sulfhydryl groups, and a decrease in both GSH and B3p expression levels was observed. A combined effect due to the glycated Hb and OS may explain what was observed in diabetic erythrocytes, while in in vitro hyperglycemia, early OS could explain B3p anion exchange capability alterations as proven by the use of melatonin. Finally, measurement of B3p anion exchange capability is a suitable tool to monitor the impact of hyperglycemia on erythrocytes homeostasis, being the first line of high glucose impact before Hb glycation. Melatonin may be useful to counteract hyperglycemia-induced OS at the B3p level.

## 1. Introduction

Diabetes mellitus type 2 (T2D) is a metabolic disease characterized by hyperglycemia, consisting of high glucose levels chronically present in the blood. According to reports by the World Health Organization (WHO), over 422 million people worldwide are affected by diabetes which is directly responsible for 1.6 million deaths each year [1]. Currently, about 10.3% of the adult population in Europe is estimated to have diabetes, and, as expected, this percentage is going to increase substantially by 2030 due to the fact of obesity and aging [2]. Oxidative stress (OS) is critically involved in diabetes pathogenesis [3] and hyperglycemia is the principal factor in the early stages of development [4]. In addition, hyperglycemic complications, such as glycosylated proteins formation [5], increase reactive oxygen species (ROS) generation, decrease nitric oxide (NO) production, and activation of the protein kinase C (PKC) and polyol pathways may contribute to OS [6]. 

During their 120 days life span, human erythrocytes are constantly exposed to glucose and other molecules present in the blood (i.e. oxidant compounds), and they have been widely investigated for their important role in different physiological conditions due to the fact of their metabolism and sensitivity to OS [7]. With regard to the effect on hyperglycemia, several studies have been performed. In this context, methylglyoxal concentration, a glucose metabolite involved in advanced glycation end-products formation (AGEs), is abnormally increased in plasma and in erythrocytes, finally exhibiting phosphatidylserine (PS) exposure on the external surface [8] and consequent eryptosis [9]. Hyperglycemia in T2D causes altered lipid–protein interactions, leading to OS and peroxidation [10] which, in turn, alters membrane lipids and membrane-bound enzyme arrangements [11]. Among the consequences of hyperglycemia, glycation of membrane proteins and the resulting decrease in protein activities have also been considered. In this regard, a typical example is provided by glycated hemoglobin (A1c), considered as a standard test to monitor glycemic status [12] since its discovery in the late 1960s. 

Oxidative damage linked to hyperglycemic conditions has also been shown to reduce the life span and rheological properties of erythrocytes, altering their shape and deformability [13] which critically correlate with Band 3 protein (B3p) function [14]. Band 3 protein, distributed in millions of copies on erythrocytes’ membranes, is responsible for Cl^−^/HCO_3_^−^ exchange through erythrocyte membranes for ion balance and gas exchange, thus reflecting erythrocytes’ homeostasis as well as homeostasis of the whole organism [15]. Band 3 protein anion exchange capability, measured by evaluating the rate constant for SO_4_^2−^ uptake, which is more easily detectable than Cl^−^ or HCO_3_^−^ exchange [14], is a good tool to verify erythrocyte function under different experimental conditions (i.e., under oxidants or toxins in vitro [16,17,18,19,20]) or in diseases associated to OS [21,22,23]. In addition, it is useful to prove the possible effects of antioxidants in preventing oxidative damage at the B3p level [24,25]. The attention paid to dietary antioxidants, influencing antioxidant defense by scavenging free radicals and reactive species [26], is important, as they are capable of counteracting OS and preventing diabetic complications [27,28]. Among antioxidants introduced by food or adopted in clinic therapy to counteract OS, melatonin (Mel) was considered in the present study [29,30]. Melatonin, a neuroendocrine hormone primarily produced by the pineal gland, has been shown to exert its antioxidant power on lipids and proteins [31], though its antioxidant activity depends on concentration and cell target [32,33]. 

Based on this evidence, the aim of the present study was to establish whether high glucose concentrations in the extracellular medium could affect the erythrocytes homeostasis via B3p alterations, as it is widely established that poorly controlled or uncontrolled diabetes leads to hyperglycemia and, consequently, to OS and glycosylation in various tissues. According to this hypothesis, the general goal was to verify whether the anion exchange capability through B3p is affected by hyperglycemia and whether the possible alteration is related to oxidative events. In this context, the following specific aims were: (1) to monitor the anion exchange capability through B3p in erythrocytes deriving from diabetic volunteers with A1c levels higher than in healthy subjects and to verify the presence of related oxidative events; (2) to monitor the anion exchange capability through B3p in in vitro hyperglycemic conditions obtained by exposing erythrocytes from healthy volunteers to increasing concentrations of glucose (15–35 mM) for 24 hours; (3) to prove the effect of OS on the anion exchange capability of B3p under in vitro-induced hyperglycemia; (4) to assay the potential antioxidant effect of Mel (100 µM) in vitro-induced hyperglycemia according to what has previously been demonstrated [32]. 

## 2. Materials and Methods 

### 2.1. Solutions and Chemicals 

The chemicals used for the present experimental protocols were purchased from Sigma (Milan, Italy). 4,4’-Diisothiocyanato-stilbene-2,2’-disulfonate (DIDS), prepared in DMSO (dimethyl sulfoxide), was diluted from 10 mM stock solution. Melatonin was prepared in 0.5% *v*/*v* ethanol and diluted from a 100 mM stock solution. The DMSO and ethanol were assayed on erythrocytes at their final concentrations to preventively exclude any damage. 

### 2.2. Erythrocytes Preparation

Blood was obtained upon orally informed consent from both healthy and diabetic volunteers. Blood samples were used after all clinical analysis were completed. Blood, collected in tubes containing anticoagulant, was washed with the following isotonic solution: 145 mM NaCl, 5 mM glucose, 5 mM HEPES (4-(2-hydroxyethyl)-1 piperazineethanesulfonic acid), pH 7.4, osmotic pressure 300 mOsm. The samples were centrifuged thrice (ThermoScientific, 1200× *g*, 5 min) to discard both plasma and buffy coat. Erythrocytes were then suspended in the isotonic solution at different hematocrit values, according to the experimental protocols reported below. Erythrocytes samples were handled according to two groups: (i) erythrocytes from diabetic volunteers (A1c > 6.5% or higher) [12]; (ii) erythrocytes withdrawn from healthy volunteers (A1c < 5.7%) [12], and successively incubated at different glucose concentrations (glucose-treated erythrocytes). With regard to this latter point, erythrocytes (3% hematocrit) were incubated for 24 h at 25 °C in isotonic solution containing different glucose concentrations, alternatively 5 mM, 15 mM, or 35 mM and then addressed to the experimental protocols.

Erythrocytes deriving from diabetic volunteers are henceforth referred to as diabetic erythrocytes, while erythrocytes deriving from healthy volunteers and then exposed for 24 h to different glucose concentrations are henceforth referred to as glucose-treated erythrocytes.

### 2.3. Osmotic Fragility in Diabetic or Glucose-Treated Erythrocytes

To verify the osmotic fragility, diabetic or glucose-treated erythrocytes, after washing in isotonic solution, were suspended at 0.5% hematocrit (isotonic solution) and then centrifuged (ThermoScientific, 25 °C, 1200× *g*, 5 min) and resuspended in a 0.7% *v*/*v* NaCl solution at 0.05% hematocrit. Hemoglobin absorbance was measured at 405 nm wavelength at different time intervals between 5 and 180 min of incubation [34,35]. Diabetic erythrocytes were directly addressed to an osmotic fragility test, while high glucose-exposed erythrocytes were assayed after 24 h incubation in high glucose solution. Analysis of blank (hemolysis solution absorbance) was also performed. 

### 2.4. SO_4_^2−^ Uptake Measurement 

#### 2.4.1. Control Condition

The SO_4_^2−^ uptake through B3p was measured as described elsewhere [21,36]. After washing, erythrocytes, derived from healthy volunteers, were suspended to 3% hematocrit in 35 mL isotonic solution containing SO_4_^2−^, henceforth called SO_4_^2−^ medium (118 mM Na_2_SO_4_, 10 mM HEPES, 5 mM glucose, pH 7.4, osmotic pressure 300 mOsm), and incubated at 25 °C. At fixed-time intervals (5–10–15–30–45–60–90–120 min), 5 mL samples of red blood cell suspensions were transferred in a tube containing DIDS (10 μM), a specific and irreversible inhibitor B3p [37], and kept on ice. At the end of incubation in SO_4_^2−^ medium, red blood cells were at least thrice washed in isotonic solution (ThermoScientific, 4 °C, 1200× *g*, 5 min) in order to eliminate SO_4_^2−^ from the external medium and then hemolysed by distilled water (1 mL). Proteins were then precipitated by perchloric acid (4% *v*/*v*). After centrifugation (4 °C, 2500× *g*, 10 min), the supernatant containing SO_4_^2−^ underwent turbidimetric analysis. The SO_4_^2−^ precipitation was performed by mixing the following components: 500 μL supernatant from each sample, 1 mL glycerol previously diluted in distilled water (1:1), 1 mL 4 M NaCl plus HCl (hydrochloric acid 37%) solution (12:1) and, finally, 500 μL 1.24 M BaCl_2_·2H_2_O. Each sample was then spectrophotometrically read at 425 nm wavelength. A calibrated standard curve previously obtained by precipitating known SO_4_^2−^ concentrations was used to convert the absorption to [SO_4_^2−^] L cells × 10^−2^. Moreover, the rate constant (min^−1^) was calculated by the equation: C_t_ = C_∞_ (1 − e^−rt^) + C_0_, where C_t_, C_∞_, and C_0_ represent the intracellular SO_4_^2−^ concentrations, respectively, at time t, 0, and ∞; *e* is the Neper number (2.7182818); *r* is the rate constant accounting for the process velocity, and *t* is the time fixed for each sample withdrawal (5–10–15–30–45–60–90–120 min). The rate constant is the time needed to reach 63% of total SO_4_^2−^ intracellular concentration [21] and [SO_4_^2−^] L cells × 10^−2^ reported in figure stands for SO_4_^2−^ micromolar concentration trapped by 10 mL erythrocytes (3% hematocrit). In a separate protocol, in order to assess that SO_4_^2−^ was effectively trapped by B3p, red blood cells were suspended in SO_4_^2−^ medium (3% hematocrit) and immediately treated with DIDS (10 µM). Then, 5 mL samples at fixed time intervals (5–10–15–30–45–60–90–120 min) were handled as described for control conditions.

#### 2.4.2. SO_4_^2−^ Uptake Measurement in Diabetic or Glucose-Treated Erythrocytes

Erythrocytes from diabetic volunteers after washing were centrifuged (ThermoScientific, 4 °C, 1200× *g*, 5 min) to replace supernatant with SO_4_^2−^ medium (3% hematocrit). The SO_4_^2−^ uptake was successively determined as described for control conditions. With regard to glucose-treated erythrocytes, SO_4_^2−^ uptake measurement was performed after 24 h incubation at different glucose concentrations by centrifuging and re-suspending samples in SO_4_^2−^ medium at 3% hematocrit.

#### 2.4.3. Preparation of Resealed Ghosts of Diabetic or Glucose-Treated Erythrocytes and SO_4_^2−^ Uptake Measurement 

Pink resealed ghosts of human erythrocytes, henceforth referred to as resealed ghosts, were prepared, as reported elsewhere [38], with slight modifications. In detail, erythrocytes from either diabetic volunteers or derived from healthy volunteers and incubated for 24 h at different glucose concentrations were washed and re-suspended in 35 mL cold hypoosmotic buffer solution (2.5 mM NaH2PO4, 5 mM HEPES, pH 7.4, 3% hematocrit). After 10 min of gently shaking at 0 °C, intracellular content and hemoglobin were discarded by several centrifugations (Beckman J2-21, 4 °C, 17,000× *g*, 20 min). Then, the supernatant was replaced by 35 mL isotonic resealing medium (145 mM NaCl, 5 mM HEPES, 5 mM Glucose, pH 7.4), previously maintained at 37 °C, and the membranes were incubated for 45 min at 37 °C to allow resealing. Finally, resealed ghosts, containing approximately 10% of the original hemoglobin content, were addressed to SO_4_^2−^ uptake measurement, according to the protocol described above for intact erythrocytes in control conditions [16]. 

### 2.5. TBARS Levels in Diabetic or Glucose-Treated Erythrocytes 

In order to prove a possible effect of lipid peroxidation on erythrocytes membrane, TBARS (thiobarbituric acid reactive species) levels, derived from the reaction between TBA (thiobarbituric acid) and MDA (malondialdehyde)—the end product of lipid peroxidation—were determined, as described by other authors [39], with slight modifications. Trichloroacetic acid (TCA, 10% *w/v* final concentration) was added to 1.5 mL of erythrocytes (either from diabetic volunteers or after incubation at different glucose concentrations) suspended at 20% hematocrit. The samples underwent centrifugation (ThermoScientific, 10 min, 3000× *g*) and 1 mL TBA (1% in 0.05 M NaOH) was added to the supernatant. Then, the mixture was heated for 30 min to 95 °C. Finally, TBARS levels were obtained by subtracting 20% of the absorbance at 453 nm from the absorbance at 532 nm (1.56 × 105 M^−1^ cm^−1^ molar extinction coefficient). The results are reported as μM TBARS levels.

### 2.6. Membrane Sulfhydryl Groups Determination in Diabetic or Glucose-Treated Erythrocytes

Concentration of membrane sulfhydryl groups was estimated by the method of Aksenov and Markesbery [40] with some modifications. Briefly, 100 μL of washed erythrocytes (either from diabetic volunteers or after incubation at different glucose concentrations) at 35% hematocrit was added to 1 mL of distilled water. Then, a 50 μL aliquot was diluted to 1 mL phosphate-buffered saline (PBS), pH 7.4, containing 1 mM ethylenediamine tetra acetic acid (EDTA). The addition of 30 μL of 10 mM 5,5’-dithiobis-(2-nitrobenzoic acid) (DTNB) started the reaction, and the samples were incubated in a dark room at 25 °C. Control samples, without protein or DTNB, were simultaneously handled. After 30 min incubation at 25 °C, the samples were spectrophotometrically read at 412 nm, and the levels of formed TNB were determined by comparison to blanks (DTNB absorbance). The results are reported as μM 3-thio-2-nitro-benzoic acid (TNB)/mg protein and data were normalized to protein content. 

### 2.7. GSH Content Measurement in Diabetic or Glucose-Treated Erythrocytes

The GSH (reduced glutathione) levels were determined on erythrocytes from diabetic volunteers or after incubation at different glucose concentrations by the method of Giustarini and collaborators [41] with some modifications. The assay is based on the oxidation of GSH by the Ellman’s reagent DTNB (5,5’-dithiobis (2-nitrobenzoic acid), producing GSSG (oxidized glutathione) and TNB absorbing at 412 nm. The levels of GSH were measured by Cayman’s GSH assay kit based on an enzymatic recycling method with glutathione reductase [17]. The amount of GSSG was calculated by the following formula: 1⁄2 GSSG = GSH_total_−GSH_reduced_, and the results are expressed as a GSH/GSSG ratio [22].

### 2.8. Measurement of Glycated Hemoglobin (A1c) in Diabetic or Glucose-Treated Erythrocytes

Glycated hemoglobin levels were determined with A1c liquidirect reagent as previously described by Sompong and collaborators [42] with slight modifications. Erythrocytes samples were lysed with hemolysis buffer (2.5 mM NaH2PO4, 5 mM HEPES, pH 7.4) and incubated with latex reagent for 5 min at 37 °C. Diabetic erythrocytes were immediately addressed to glycated hemoglobin measurement, while high glucose-exposed erythrocytes were assayed after 24 h incubation in high glucose solution. The absorbance was measured at 610 nm and A1c levels calculated from a standard curve using A1c and expressed as % A1c.

### 2.9. Erythrocytes Membranes Preparation and SDS-PAGE in Diabetic or Glucose-Treated Erythrocytes

Erythrocyte membranes were prepared as described by other authors [43] with slight modifications. Briefly, packed erythrocytes, derived from either diabetic volunteers or after incubation at different glucose concentrations, were diluted into 1.5 mL of 2.5 mM NaH2PO4 (cold hemolysis solution) containing a cocktail of protease and phosphatase inhibitors (1 mM PMSF, 1 mM NaF, 1 mM Na3VO4). Samples were centrifuged several times (Eppendorf, 4 °C, 13,000× *g*, 10 min) to remove hemoglobin. The obtained membranes were solubilized by SDS (sodium dodecyl sulfate, 1% *v*/*v*) and kept for 20 min on ice. Samples were then centrifugated (Eppendorf, 4 °C, 13,000× *g*, 30 min), and the supernatant containing solubilized membrane proteins was used for determination of protein content [44] and stored at 80 °C until use. After thawing, the membranes derived from each experimental condition were solubilized in Laemmli Buffer (1:1 volume ratio) [45], heated at 95 °C for 10 min, and finally the 20 μL of proteins were loaded. The samples were separated on 7.5% polyacrylamide gel before they were transfered to a PVDF (polyvinylidene fluoride) membrane. 

### 2.10. Western Blot Analysis 

The membranes were incubated overnight at 4° C with monoclonal anti-Band 3 protein antibody (B9277, 1:5000, Sigma–Aldrich, Milan, Italy), produced in mouse and diluted in Tris-Buffer (TBS) with 5% bovine serum albumin (BSA) and 0.1% Tween 20. Successively, membranes were incubated for 1 h with peroxidase-conjugated goat anti-mouse IgG secondary antibodies (A9044, 1:10,000, Sigma–Aldrich, Milan, Italy) at room temperature. To assess the presence of equal amounts of protein, monoclonal anti-Actin antibody (A1978, 1:1000, Sigma–Aldrich, Milan, Italy) produced in mouse were incubated on the same membranes by using a stripping method, thus allowing to detect different protein targets within a single membrane [46]. A chemiluminescence detection system (Super Signal West Pico Chemiluminescent Substrate, Pierce Thermo Scientific, Rockford, IL, USA) was used to detect signals, and images of them were imported to analysis software (Image Quant TL, v2003). The relative expression of protein bands was determined by densitometry (Bio-Rad ChemiDocTM XRS+). 

### 2.11. Experimental Data and Statistics 

Data are expressed as arithmetic means ± SEM for statistical analysis performed by GraphPad Prism software (version 5.00 for Windows; San Diego, CA). Significant differences among means were tested by one-way analysis of variance (ANOVA), followed by Bonferroni’s multiple comparison post-hoc test or Student’s *t*-test (paired values). Statistically significant differences were assumed at *p* < 0.05; *n* represents the number of independent experiments.

## 3. Results

### 3.1. Diabetic Erythrocytes

#### 3.1.1. Osmotic Fragility Measurement 

**Figure 1:**
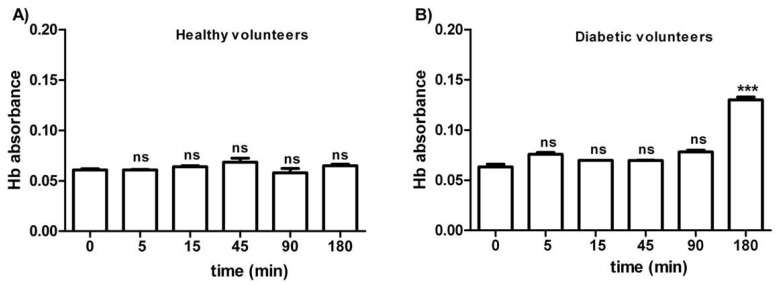
Osmotic fragility measured as hemoglobin (Hb) absorbance (optical density) in erythrocytes from healthy volunteers (**A**) and diabetic volunteers (**B**). *ns* (not significant) versus different time intervals considered (5–15–45–90 min); *** *p* < 0.05 versus different time intervals (0–5–15–45–90 min) as determined by one-way ANOVA followed by Bonferroni’s post-hoc test (*n =* 10).

Figure 1 shows the osmotic fragility in erythrocytes from both healthy (A) and diabetic volunteers (B), reported as absorbance of hemoglobin released at different times of incubation (0–5–15–45–90–180 min) in a 0.7% *v*/*v* NaCl solution. As depicted, the osmotic fragility of erythrocytes from both groups was not significantly different with respect to hemoglobin levels at the following time intervals: 0–5–15–45–90 min. With regard to erythrocytes from diabetic volunteers, osmotic fragility at 180 min was significantly higher than all values at the other time intervals (0–5–45–90 min).

#### 3.1.2. SO_4_^2−^ Uptake Measurement 

**Figure 2:**
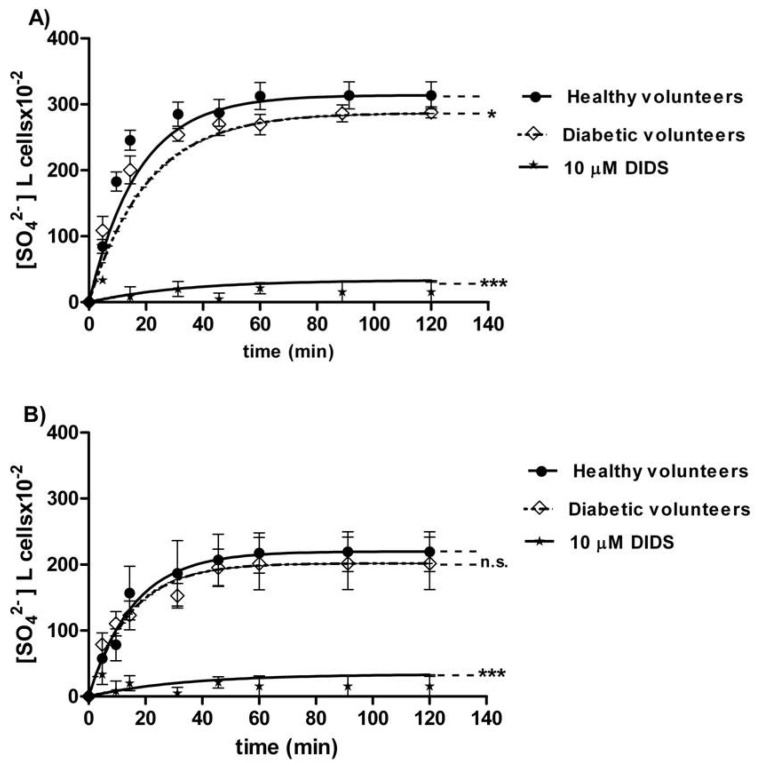
(**A**) Time course of SO_4_^2−^ uptake in healthy volunteers (control) and in erythrocytes from diabetic volunteers. ^*^ *p* < 0.05, and *** *p* < 0.001 significant versus control as determined by one-way ANOVA followed by Bonferroni’s post-hoc test (*n* = 16). (**B**) Time course of SO_4_^2−^ uptake in resealed ghosts from healthy volunteers (control) and from diabetic volunteers. *ns* (not significant) versus control; *** *p* < 0.001 significant versus control as determined by one-way ANOVA followed by Bonferroni’s post-hoc test (*n* = 16).

The curves depicted in Figure 2A describe SO_4_^2−^ uptake as a function of time in erythrocytes from healthy (control) and diabetic volunteers. In control conditions, the rate of SO_4_^2−^ uptake was augmented until equilibrium within 45 min with a rate constant value of 0.063 ± 0.001 min^−1^. The rate constant in erythrocytes from diabetic volunteers (0.113 ± 0.001 min^−1^, ^*^ *p* < 0.05, Table 1) was significantly higher than the control. The exposure to 10 µM DIDS since the beginning of incubation in SO_4_^2−^ medium inhibited SO_4_^2−^ uptake (0.018 ± 0.001 min^−1^, *** *p* < 0.001, Table 1). The SO_4_^2−^ quantity internalized by red blood cells from diabetic volunteers at 45 min of incubation in SO_4_^2−^ medium (269.84 ± 16.8) was not significantly different with respect to that one measured in control cells (287.24 ± 20.3, Table 1). In DIDS-treated cells, intracellular SO_4_^2−^ quantity at 45 min of incubation (4.75 ± 8.50) was significantly lower than those determined in both the control and in erythrocytes from diabetic volunteers (*** *p* < 0.001, Table 1).

#### 3.1.3. Resealed Ghosts 

The curves depicted in Figure 2B describe SO_4_^2−^ uptake in resealed ghosts from healthy (control) and diabetic volunteers. In the control resealed ghosts, SO_4_^2−^ uptake increased until equilibrium within 45 min, exhibiting a rate constant of 0.063 ± 0.001 min^−1^. The rate constant in resealed ghosts from diabetic volunteers (0.067 ± 0.002 min^−1^, Table 2) was not significantly different from the control. The exposure to 10 µM DIDS applied at the beginning of incubation in SO_4_^2−^ medium obscured SO_4_^2−^ uptake (0.017 ± 0.001 min^−1^, *** *p* < 0.001, Table 2). The SO_4_^2−^ amount internalized by resealed ghosts from diabetic volunteers at 45 min of incubation in SO_4_^2−^ medium (197.18 ± 28) was not significantly different with respect to the one measured in control ghosts (287.24 ± 20.3, Table 2). In DIDS-treated ghosts, intracellular SO_4_^2−^ at 45 min of incubation (21.45 ± 8.50) was significantly lower than those determined in both the control resealed ghosts and the resealed ghosts from diabetic volunteers (*** *p* < 0.001, Table 2).

#### 3.1.4. Oxidative Conditions Assessment 

**Figure 3:**
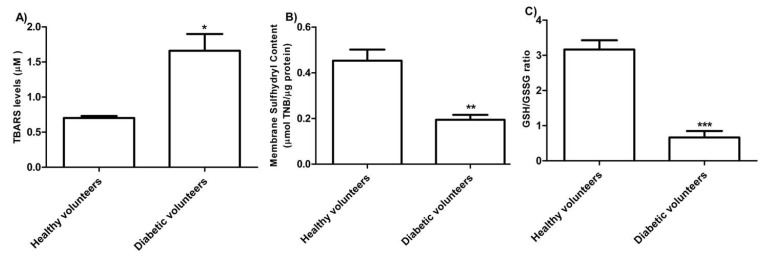
(**A**) TBARS levels (µM) in healthy volunteers (control) and in diabetic volunteers. ^*^ *p* < 0.05 significant versus control as determined by the Student’s *t*-test (*n* = 8). (**B**) Membrane sulfhydryl content measured as µmol TNB/µg protein in healthy volunteers (control) and diabetic volunteers. ^**^ *p* < 0.01 significant versus control as determined by the Student’s *t*-test (*n* = 8). (**C**) Estimation of the GSH/GSSG ratio in erythrocytes from healthy volunteers (control) and diabetic volunteers. *** *p* < 0.001 significant versus control as determined by the Student’s *t*-test (*n* = 8).

The levels of TBARS estimated in erythrocytes from diabetic volunteers were significantly higher than those detected in erythrocytes from healthy volunteers (control) (Figure 3 A, ^*^ *p* < 0.05). As depicted in Figure 3B, the membrane sulfhydryl content measured in erythrocytes from diabetic volunteers was significantly lower than the one measured in erythrocytes from healthy volunteers (control) (^**^ *p* < 0.01) as well as the GSH/GSSG ratio (Figure 3 C) (*** *p* < 0.001). 

#### 3.1.5. Band 3 Protein Expression Levels Determination 

**Figure 4:**
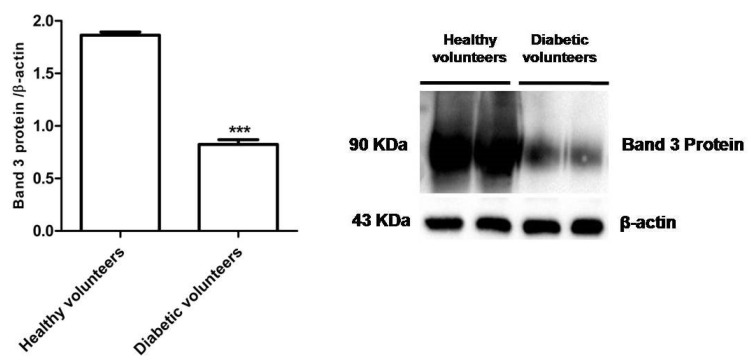
Expression levels of Band 3 protein measured in erythrocytes from healthy volunteers (control) or in diabetic volunteers detected by Western Blot analysis. *** *p* < 0.001 versus control as determined by the Student’s *t*-test (*n* = 5).

Figure 4 shows that B3p levels in erythrocytes from diabetic volunteers were significantly lower (*** *p* < 0.001) than those determined in erythrocytes from healthy volunteers (control).

### 3.2. Glucose-Treated Erythrocytes

#### 3.2.1. Osmotic Fragility Measurement 

**Figure 5:**
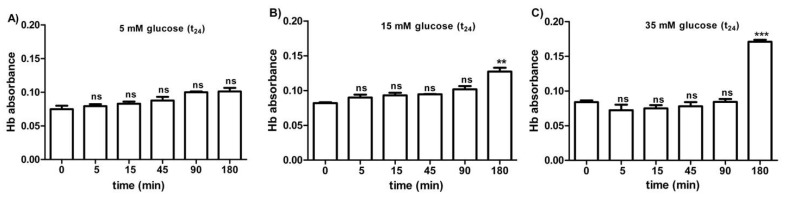
Osmotic fragility measured as hemoglobin (Hb) absorbance (optical density) in erythrocytes after treatment for 24 h with 5 mM glucose (t_24_) (**A**), 15 mM glucose (t_24_) (**B**), and 35 mM glucose (t_24_) (**C**). *ns* (not significant) versus different time intervals (5–15–45–90–180 min); ^**^ *p* < 0.01 and *** *p* < 0.001 versus different time intervals at 15 mM glucose (t_24_) and 35 mM glucose (t_24_) as determined by one-way ANOVA followed by Bonferroni’s post-hoc test (*n* = 10).

Figure 5A–C shows the osmotic fragility reported as hemoglobin absorbance in erythrocytes treated for 24 h with different glucose concentrations (5–15–35 mM). At different times (0–5–15–45–90 min) of incubation in 0.7% *v*/*v* NaCl solution, the osmotic fragility was not significantly different with respect to what was observed at the beginning (0 min) of the test at each glucose concentration considered. After 180 min of incubation with either 15 or 35 mM glucose, respectively, (Figure 5B,C), the osmotic fragility was significantly higher (^**^p < 0.05; *** *p* < 0.001) than what was observed at the other time intervals (0–5–45–90 min).

#### 3.2.2. SO_4_^2−^ Uptake Measurement 

**Figure 6:**
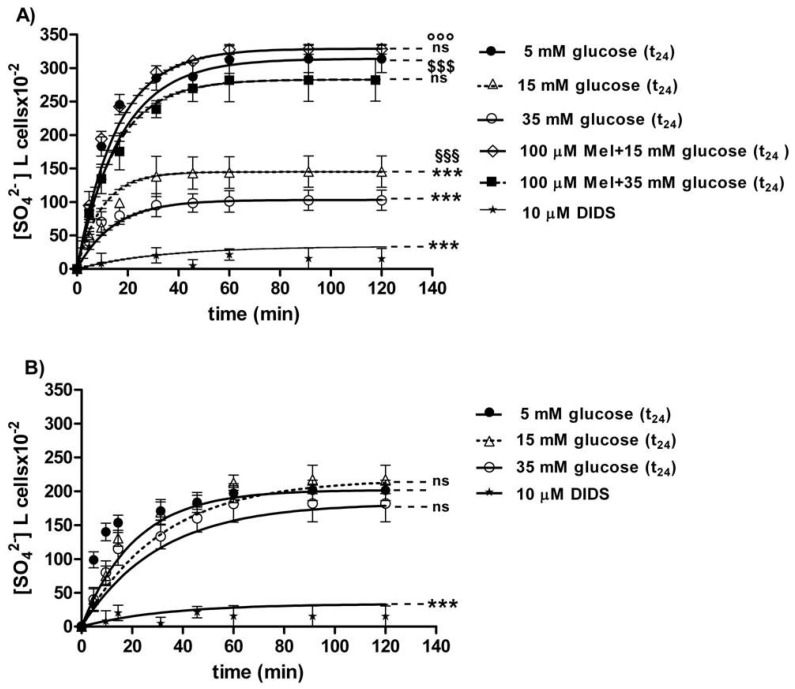
(**A**) Time course of SO_4_^2−^ uptake in glucose-treated erythrocytes at different glucose concentrations (5–15–35 mM) incubated for 24 h. *ns* versus 5 mM glucose (control); *** *p* < 0.001 versus control; ^§§§^ *p* < 0.001 significant versus 35 mM glucose;^°°°^ *p* < 0.001 significant versus 15 mM glucose and ^$$$^ *p* < 0.001 significant versus 35 mM glucose as determined by one-way ANOVA followed by Bonferroni’s post-hoc test (*n* = 16). (**B**) Time course of SO_4_^2−^ uptake in resealed ghosts from glucose-treated erythrocytes incubated for 24 h at different glucose concentrations (5–15–35 mM). *** *p* < 0.001 versus 5 mM glucose (t_24_) (control) and *ns* versus control as determined by one-way ANOVA followed by Bonferroni’s post-hoc test (*n* = 16).

The curves depicted in Figure 6A describe SO_4_^2−^ uptake as a function of time. In particular, the rate constant for SO_4_^2−^ uptake increased until equilibrium (45 min) with a value of 0.057±0.001 min^−1^ in 5 mM glucose (t_24_) erythrocytes (control) (Table 1). Erythrocytes treated with 15 mM glucose(t_24_) and 35 mM glucose(t_24_) exhibited a rate constant of, respectively, 0.109 ± 0.001 min^−1^ and 0.129 ± 0.001 min^−1^, both significantly higher than the one measured in the control conditions (*** *p* < 0.001, Table 1). Pre-exposure to 100 µM Mel significantly impaired such an increase, as a rate constant of 0.065 ± 0.001 min^−1^ and 0.061 ± 0.001 min^−1^ was, respectively, observed (^°°°^ *p* < 0.001 and ^§§§^ *p* < 0.001, Table 1) in 15 mM and 35 mM glucose-treated erythrocytes. The exposure to 10 µM DIDS applied at the beginning of incubation in SO_4_^2−^ medium impaired SO_4_^2−^ uptake (0.018 ± 0.001 min^−1^, *** *p* < 0.001, Table 1).

The SO_4_^2−^ quantity internalized by red blood cells treated with 15 mM glucose (t_24_) and 35 mM glucose(t_24_) at 45 min of incubation in SO_4_^2−^ medium (98.42 ± 13.75; 143.15 ± 24) was significantly lower than the one measured in the control cells (287.24 ± 18.20) (*** *p* < 0.001 and ^**^ *p* < 0.01, Table 1). Moreover, SO_4_^2−^ quantity internalized by erythrocytes treated with 100 µM Mel plus either 15 mM or 35 mM glucose was significantly higher (310.47 ± 17.7 and 269.76 ± 19.7) than that one measured at 15 mM glucose (t_24_) and 35 mM glucose (t_24_) alone (^°°°^ *p* < 0.001 and ^§§§^ *p* < 0.001, Table 1).

In DIDS-treated cells, the intracellular SO_4_^2−^ quantity after 45 min incubation (4.75 ± 8.50) was significantly lower than that one determined in control erythrocytes (*** *p* < 0.001, Table 1).

#### 3.2.3. Resealed Ghosts 

The curves depicted in Figure 6B describe SO_4_^2−^ uptake as a function of time in resealed ghosts from glucose-treated erythrocytes. In control resealed ghosts, SO_4_^2−^ uptake increased until equilibrium (45 min), exhibiting a rate constant of 0.063±0.001 min^−1^. The rate constant values of 0.065 ± 0.001 min^−1^ and 0.066 ± 0.001 min^−1^ observed in resealed ghosts at, respectively, 15 mM glucose (t_24_) and 35 mM glucose (t_24_) were not significantly different with respect to control (Table 2). The exposure to 10 µM DIDS applied at the beginning of incubation in SO_4_^2−^ medium inhibited SO_4_^2−^ uptake (0.017 ± 0.001 min^−1^, *** p < 0.001, Table 2).

The SO_4_^2−^ quantity internalized after 45 min incubation in SO_4_^2−^ medium by resealed ghosts of erythrocytes previously treated with 15 mM (t_24_) and 35 mM glucose (t_24_) (160.3 ± 20.6 and 183.5 ± 14.6) was not significantly different with respect to the one measured in control ghosts (183.69 ± 24, Table 2). In DIDS-treated ghosts, intracellular SO_4_^2−^ quantity after 45 min incubation (21.45 ± 8.50) was significantly lower than those determined in both control resealed ghosts and diabetic volunteer resealed ghost erythrocytes (*** *p* < 0.001, Table 2).

#### 3.2.4. Oxidative Conditions Assessment 

**Figure 7:**
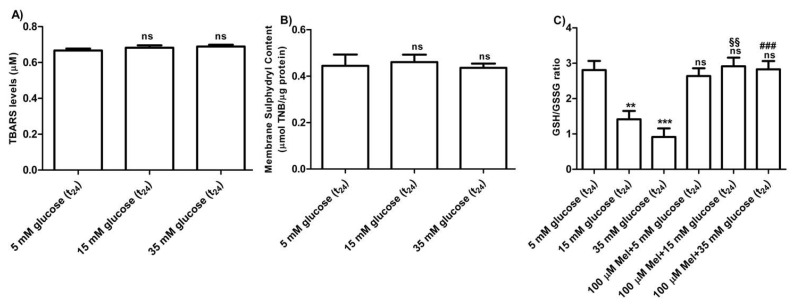
(**A**) TBARS levels (µM) measured in glucose-treated erythrocytes incubated for 24 h at different glucose concentrations (5–15–35 mM). *ns* (not significant) versus 5 mM glucose (t_24_) (control) as determined by Bonferroni’s post-hoc test (*n* = 8). (**B**) Membrane sulfhydryl content measured as µmol TNB/µg protein in glucose-treated erythrocytes (5–15–35 mM glucose) incubated for 24 h. *ns* (not significant) versus control by Bonferroni’s post-hoc test (n = 8). (**C**) Estimation of the GSH/GSSG ratio measured in glucose-treated erythrocytes at different concentrations (5–15–35 mM glucose) incubated for 24 h. *ns* (not significant) versus control; *** *p* < 0.001 significant versus control; ^§§^ *p* < 0.01 versus 15 mM glucose and ^###^ *p* < 0.001 significant versus 35 mM glucose as determined by one-way ANOVA followed by Bonferroni’s post-hoc test (*n* = 8).

The levels of TBARS determined in erythrocytes treated with different concentrations of glucose (15–35 mM) were not significantly different with respect to those detected in 5 mM glucose (t_24_) treated erythrocytes (control) (Figure 7A). As depicted in Figure 7B, membrane sulfhydryl content measured in glucose-treated erythrocytes was not significantly different with respect to that measured in 5 mM (t_24_) glucose-treated erythrocytes (control). Moreover, the GSH/GSSG ratio (Figure 7C) measured in 15 mM and 35 mM (t_24_) glucose-treated erythrocytes was significantly lower (*** *p* < 0.001; ** *p* < 0.01) than what measured at 5 mM glucose (t_24_) (control). Pre-exposure to 100 µM Mel significantly impaired this reduction (^§§^ *p* < 0.01, ^###^ *p* > 0.001) in both 15 mM and 35 mM glucose-treated erythrocytes.

#### 3.2.5. Glycated Hemoglobin (A1c) Measurement 

**Figure 8:**
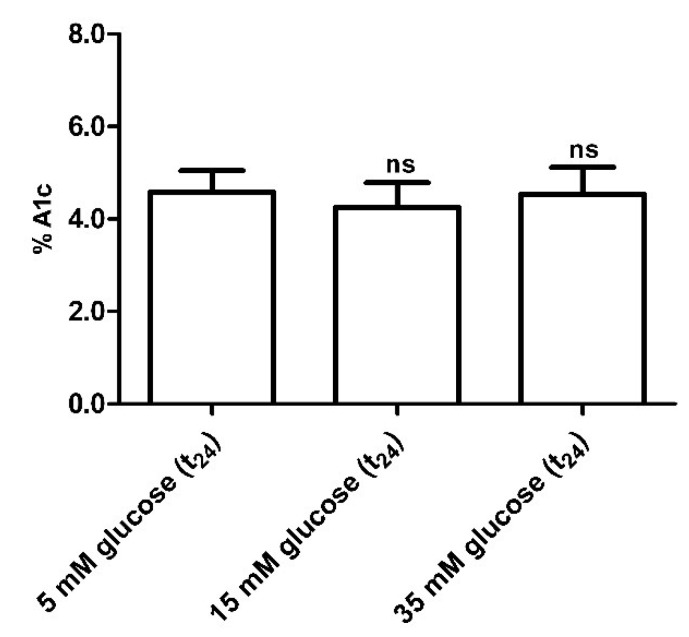
Glycated hemoglobin measurement in glucose-treated erythrocytes incubated for 24 h at different glucose concentrations (5–15–35 mM). *ns* (not significant) versus 5 mM glucose (t24) (control) as determined by one-way ANOVA followed by Bonferroni’s post-hoc test (*n* = 10).

Glycated hemoglobin levels in red blood cells exposed for 24 h to different glucose concentrations (15–35 mM) were not significantly different with respect to those detected in 5 mM (t_24_) glucose-treated erythrocytes (control) (Figure 8). 

#### 3.2.6. Band 3 Protein Expression Levels Determination 

**Figure 9:**
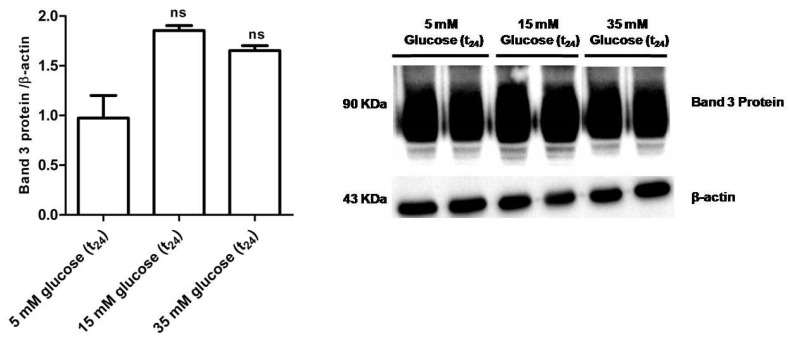
Band 3 protein expression levels measured in glucose- treated erythrocytes incubated for 24 h at different glucose concentrations (5–15–35 mM), detected by Western blot analysis. *ns* versus 5 mM glucose (t_24_) (control) as determined by one-way ANOVA followed by Bonferroni’s post-hoc test (*n* = 5).

Figure 9 shows that B3p levels in erythrocytes incubated with either 15 mM glucose (t_24_) or 35 mM glucose (t_24_) were not significantly different with respect to those determined in erythrocytes treated with 5 mM glucose (t_24_) (control). 

## 4. Discussion

Chronic hyperglycemia is a major factor in diabetic complications development. Clinical evidence and experimental data support the hypothesis that reactive oxygen species (ROS) generation is augmented in T2D and that diabetes development is strictly related to OS with the mechanisms still unclear. Due to the fact of their sensitivity to oxidation, red blood cells represent a unique model to study OS and to possibly verify the efficacy of antioxidant therapies [20] and, in this context, the role of membrane transport systems in erythrocytes from diabetic patients has already been investigated. Some authors reported about Ca_2_^+^-ATPase activity of erythrocyte membranes decreased in both diabetes [47,48] and in erythrocytes treated ex vivo with glucose [49], probably due to the glycation of Ca_2_^+^-ATPase [50,51].

Less is known about B3p’s function, essential to erythrocytes homeostasis, the assessment of which has already been proven as a good tool to reveal damage in the case of OS-related diseases, such as systemic scleroderma and canine leishmaniasis [22,23], or in other in vitro oxidative conditions [16,32]. 

Damage due to the fact of OS in hyperglycemic conditions has already been shown to reduce rheological properties, erythrocytes survival, and to affect erythrocytes’ shape [13] which is known to critically correlate with B3p function [16,32,52]. 

On this premise, the present paper aimed to verify whether anion exchange capability through B3p is compromised in erythrocytes from diabetic volunteers or, ex vivo, after 24 h exposure to high glucose concentrations (15–35 mM) in accordance with what was reported by other authors [42].

As an increase of A1c levels is commonly associated to T2D, erythrocytes from diabetic volunteers was considered. Glycated hemoglobin, a minor fraction of Hb, is in vivo produced by non-enzymatic binding of glucose to N-terminal amino acids of Hb A beta chains [53,54]. 

The first step of the present study was to assess erythrocytes’ integrity before conducting measurement of anion exchange capability through B3p. With this purpose, erythrocytes from diabetic volunteers underwent an osmotic fragility test. As no significant release of Hb was seen after 120 min of incubation in a 0.7% *v*/*v* NaCl solution, SO_4_^2−^ uptake measurement was performed. According to what was reported by other groups [42,55], osmotic fragility tests showed that diabetic erythrocytes were more susceptible to hemolysis than those of Healthy volunteers (Figure 1A,B) after 180 min of incubation in 0.7% *v*/*v* NaCl solution, probably due to the presence of glycated Hb.

Coming back to the assessment of anion exchange capability through B3p, the rate constant for SO_4_^2−^ uptake was increased in red blood cells from diabetic volunteers (Figure 2A). To better clarify the mechanisms underlying such acceleration, the technique of resealed ghosts was employed [16]. The evidence that the rate constant for SO_4_^2−^ uptake of hemoglobin-free resealed ghosts from diabetic erythrocytes is similar to that one of the intact non-diabetic erythrocytes could suggest that glycation of Hb may affect its cross-link with membrane proteins, namely, B3p, with consequences on anion exchange (Figure 2B). 

As expected, the altered anion exchange capability through B3p in erythrocytes from diabetic volunteers was also associated to an increase of intracellular OS, as proven by the loss of membrane asymmetry via formation of thiobarbituric acid reactive substances, oxidation of sulfhydryl groups of membrane proteins, and a decrease in the GSH/GSSG ratio (Figure 3A–C) [56]. Furthermore, a decrease in B3p expression levels was also observed (Figure 4). An increase in lipid peroxidation, a decrease in membrane sulfhydryl groups, and reduced B3p expression levels have previously been associated to a reduced rate of anion exchange capability after exposure to H_2_O_2_-induced oxidative stress [16]. In this regard, as the present findings show an accelerated rate constant for SO_4_^2−^, the alteration should be ascribed to glycation rather than oxidative damage, though both conditions are associated to diabetes [5,6]. 

To complete the frame, in an attempt to further focus on the impact of hyperglycemia on anion exchange capability through B3p and to possibly explain why the rate constant for SO_4_^2−^ uptake is accelerated in red blood cells exposed to high glucose, hyperglycemia was modeled in vitro and experiments were performed by exposing erythrocytes of healthy volunteers to high glucose levels which mimics early hyperglycemic conditions [55]. Basically, transport via GLUT1 (insulin-independent glucose transporter) makes glucose concentration in red blood cells cytosol similar to plasmatic levels which are normally approximately 5 mM. Nevertheless, such values may increase under hyperglycemia conditions [57]. 

With regard to such in vitro tests, high glucose concentrations (15 and 35 mM) applied for 24 h make erythrocytes more susceptible to osmotic fragility, similar to what was observed in erythrocytes from diabetic volunteers, i.e., after at least 180 min of incubation in a 0.7% *v*/*v* NaCl solution (Figure 5B,C). Such alteration is also associated to a significant change in anion exchange capability through B3p observed after 24 h incubation at either 15 or 35 mM glucose (Figure 6A). To explain why the rate constant for SO_4_^2−^ was accelerated in such treated erythrocytes, resealed ghosts were used, and, in addition, oxidative damage and B3p expression levels were monitored, similarly to what was performed for diabetic erythrocytes.

The first finding was that, similar to what was assessed in diabetic erythrocytes, when erythrocytes were deprived of Hb, the rate constant for SO_4_^2−^ totally recovered, suggesting also in this case that 24 h incubation at high glucose concentrations may induce Hb alterations, reflecting on B3p function (Figure 6 B). Similar to the first experimental frame of this work, the impact of oxidative conditions possibly associated to in vitro hyperglycemia was monitored. Our results show that only the GSH/GSSG ratio changed as detected in diabetic erythrocytes, while there were no oxidative membrane sulfhydryl group level or lipid peroxidation events (Figure 7A–C), neither was there a decrease in B3p expression levels associated to the observed accelerated rate constant for SO_4_^2−^ uptake (Figure 9). In an attempt to verify whether Hb glycation was involved in this response—based on what was obtained with hemoglobin-free resealed ghosts in studies by other authors [42,58]—the achievement of Hb glycation after 24 h incubation of erythrocytes with 45 mM glucose (lower than glucose concentration here used) was considered. As our results demonstrate, after 24 h exposure to 15 or 35 mM glucose erythrocytes did not show Hb glycation (Figure 8); the alteration in anion exchange capability through B3p could be ascribed to early oxidative damage (shown only by changed GSH levels) rather than to autooxidation leading to glycation of proteins, as shown by the abovementioned studies [42,58]. Such discrepancy may be due to the different glucose concentration employed. 

To better support this finding, an antioxidant compound, possibly counteracting the effect of in vitro hyperglycemia, was used. In this regard, Mel has been used on the basis of what was recently reported by our group [32].

The exposure of red blood cells to Mel prevented the increase in the rate constant for SO_4_^2−^ uptake observed under high glucose treatment (15 and 35 mM), demonstrating a correlation between Mel, anion exchange capability through B3p, and hyperglycemia in an in vitro model (Figure 6A). This adds new elements to a previous investigation recently performed by our group [32] about the antioxidant effects of Mel. In addition, such a result is in accordance with other authors, reporting that Mel restores the activity of antioxidant enzymes in red blood cells from multiple sclerosis patients which confirms that under OS some antioxidant enzymes of erythrocytes may be selectively activated [59]. In line with these authors, the present data describe Mel’s beneficial effect revealed by the GSH/GSSG ratio restoration observed after pre-exposure to this antioxidant. Hence, it could be suggested that oxidative damage by high glucose concentrations could be mainly due to the alterations at the endogen antioxidant system level, namely, intracellular GSH, rather than to an effect directly exerted at the membrane level. Such findings do not seem in line with what was previously demonstrated in an H_2_O_2_-induced oxidative stress model on human erythrocytes [32] as different mechanisms of action of oxidative damage could occur. 

## 5. Conclusions

In conclusion, the present results suggest that: (i) the B3p function assessment is a sensitive tool to assess the impact of hyperglycemia on red blood cells’ homeostasis, namely, in subjects with A1c levels not associated to diabetes but possibly exhibiting a risk of poorly controlled glycemic levels; (ii) in non-diabetic subjects, changes in anion exchange capability through B3p may be one of the first consequences of hyperglycemia; (iii) hyperglycemia complications may be related to antioxidant systems reflecting on B3p function, in addition to glycation of proteins; (iv) in diabetes, the damage at the B3p level is due to the Hb glycation and is exacerbated by OS. Finally, the use of Mel in the in vitro model contributed to: (1) supporting the hypothesis of oxidative damage at the B3p level in early hyperglycemia conditions; (2) adding more knowledge about its antioxidant power, especially in the case of OS associated to early hyperglycemic conditions; and (3) confirming that homeostasis of red blood cells can be protected by Mel against OS.

## Figures and Tables

**Table 1:** Rate constant for SO_4_^2−^ uptake and SO_4_^2−^ quantity trapped in both diabetic and glucose-treated erythrocytes. Data are presented as means ± SEM from separate *n* experiments, where *ns* versus 5 mM glucose (t_24_) (control) and healthy volunteers; ^*^ *p* < 0.05 versus healthy volunteers; ^**^ *p* < 0.001 versus control; *** *p* < 0.001 versus control; ^§§§^ *p* < 0.001 significative different versus 35 mM glucose (t_24_);^*°°°*^ *p* < 0.001 significant versus 15 mM glucose (t_24_) and ^$$$^ *p* < 0.001 significant versus 35 mM glucose (t_24_) as determined by one-way ANOVA followed by Bonferroni’s post-hoc test.

	Rate Constant min^−1^	Time min	*N*	SO_4_^2−^ Quantity Trapped at 45 min of Incubation in SO_4_^2−^ Medium [SO_4_^2−^] L cells × 10^−2^
Healthy volunteers	0.063 ± 0.001	16	16	287.24 ± 20.3
Diabetic volunteers	0.113 ± 0.001 *	9	16	269.84 ± 16.8 ^ns^
5 mM glucose (t_24_)	0.057 ± 0.001	17	16	287.24 ± 18.20
15 mM glucose (t_24_)	0.109 ± 0.001 ***^, §§§^	9	16	98.42 ± 13.75 ***
35 mM glucose (t_24_)	0.129 ± 0.001 ***	7	16	143.15 ± 24 **
100 µM Mel + 15 mM glucose	0.065 ± 0.001 ***	15	16	310.47 ± 17.7 °°°
100 µM Mel + 35 mM glucose	0.061 ± 0.001 ^ns, $$$^	16	16	269.76 ± 19.7 ^$$$^
10 µM DIDS	0.018 ± 0.001 ***	55	10	4.75 ± 8.50 ***

**Table 2:** Rate constant for SO_4_^2−^ uptake and quantity of SO_4_^2−^ trapped in both resealed ghosts of glucose-treated erythrocytes and in resealed ghosts from diabetic volunteers. Data are presented as means ± SEM from separate *n* experiments, where *ns* versus 5 mM glucose (t_24_) and healthy volunteers (control);*** *p* < 0.001 versus control as determined by one-way ANOVA followed by Bonferroni’s post-hoc test.

	Rate Constant min^−1^	Time min	*N*	SO_4_^2−^ Quantity Trapped at 45 min of Incubation in SO_4_^2−^ Medium [SO_4_^2−^] L cells × 10^−2^
Healthy volunteers	0.063 ± 0.001	16	16	207.01 ± 38
Diabetic volunteers	0.067 ± 0.002 ^ns^	15	16	197.18 ± 16.8 ^ns^
5 mM glucose (t_24_)	0.064 ± 0.001	16	16	183.69 ± 24
15 mM glucose (t_24_)	0.065 ± 0.001 ^ns^	15	16	160.3 ± 20.06 ^ns^
35 mM glucose (t_24_)	0.066 ± 0.001 ^ns^	15	16	183.15 ± 14.6 ^ns^
10 µM DIDS	0.017 ± 0.001 ***	58	10	21.45 ± 8.50 ***
